# Coffee as a Source of Antioxidants and an Elixir of Youth

**DOI:** 10.3390/antiox14030285

**Published:** 2025-02-27

**Authors:** Zofia Kobylińska, Marek Biesiadecki, Ewelina Kuna, Sabina Galiniak, Mateusz Mołoń

**Affiliations:** 1 Faculty of Biology and Nature Protection, Rzeszów University, Zelwerowicza 4, 35-601 Rzeszów, Poland; zosiakob@icloud.com (Z.K.); ekuna@ur.edu.pl (E.K.); 2 Faculty of Medicine, Rzeszów University, Warzywna 1a, 35-310 Rzeszów, Poland; mbiesiadecki@ur.edu.pl

**Keywords:** antioxidants, anti-aging, coffee, polyphenols

## Abstract

Coffee is more than a universally loved beverage; it is a complex matrix of bioactive compounds that contribute to its multifaceted health benefits. From its role as a potent source of antioxidants to its potential anti-aging effects, coffee has proven to be a valuable component of a balanced diet. This paper highlights the extensive scientific evidence supporting coffee’s ability to combat oxidative stress, enhance cognitive function, and improve metabolic and cardiovascular health. Additionally, its role in modulating key cellular pathways underscores its potential to positively influence aging and longevity. This manuscript emphasizes coffee’s broader cultural, economic, and historical significance, illustrating its enduring relevance in contemporary society. Despite minor discrepancies in research findings, the preponderance of evidence underscores coffee’s potential as a functional food with profound implications for healthspan and aging. While promising, translating findings to humans requires further clinical research.

## 1. Introduction

Coffee, a beloved beverage enjoyed by millions worldwide, is celebrated not only for its rich flavor and invigorating aroma but also for its many health benefits. Research shows that regular coffee consumption can boost cognitive function, enhancing alertness, concentration, and overall mental performance [1]. Additionally, coffee is a rich source of antioxidants, which help combat oxidative stress (OS) and lower the risk of chronic diseases, including heart disease and cancer. Its bioactive compounds, such as caffeine and polyphenols, contribute to its anti-inflammatory properties, aiding in the management of inflammation-related conditions. Furthermore, studies have associated coffee consumption with a reduced risk of neurodegenerative diseases like Alzheimer’s and Parkinson’s [2,3]. Moreover, coffee has been found to support metabolic health by improving insulin sensitivity and reducing the risk of type 2 diabetes. Regular coffee drinkers may also experience a reduced risk of stroke and liver diseases, including liver cancer and cirrhosis [4]. The beverage’s potential anti-aging effects are another area of interest, with some studies suggesting that coffee can promote longevity and improve overall healthspan [5]. Furthermore, coffee’s role in enhancing physical performance and endurance makes it a popular choice among athletes. Overall, the diverse health benefits of coffee make it a valuable addition to a balanced diet and a subject of ongoing scientific research. This paper aims to compile the latest data on the history and composition of coffee, its antioxidant properties, and the anti-aging benefits of coffee consumption.

### 1.1. The Role of Coffee in Contemporary Society

Coffee holds a vital place in modern society, serving not only as a widely consumed beverage but also as a cornerstone of social and cultural life. It has become an integral part of daily routines and a symbol of community and connection. Beyond its role as a drink, coffee is deeply embedded in cultural traditions and diverse customs worldwide. Its importance extends further into the global economy, as coffee ranks among the most traded commodities, highlighting its economic significance. With billions of people consuming coffee daily, it stands as one of the most popular beverages globally. In the 2022/2023 period alone, global coffee consumption reached 168.2 million 60-kg bags, and this figure continues to rise annually [6,7].

### 1.2. Short History of Coffee

*Coffea arabica* is one of the original coffee species, naturally occurring in regions of Africa and native to Arabia [8]. There is limited historical documentation regarding the use of coffee as a beverage before the Islamic era [9]. Legends attribute the discovery of coffee’s stimulating properties to two figures. One story tells of Ethiopian goat herder Kaldi, who noticed his goats became energetic after eating coffee berries and tried them himself. Another legend, recorded in a 1699 manuscript by Antoine Galland, credits Sheikh Omar, who, while exiled in 1258, brewed a decoction from coffee berries and leaves to curb hunger, uncovering its effects [9].

The use of coffee dates back to the 9th century in Ethiopia, where it was likely consumed in its unprocessed form. Before the year 1000, Ethiopian tribes ground ripe coffee berries and mixed them with animal fat to create an energizing snack [7,9]. However, with the discovery of the roasting process in the 14th century, coffee gained prominence across the Arabian Peninsula, eventually spreading to Mecca and Medina by the late 15th century [7,8]. By around 1540, coffee had reached Cairo, Damascus, and Aleppo. Subsequently, around 1554–1555, it arrived in Istanbul, which became the unofficial capital of coffee, where the first coffeehouses emerged. By the end of the 16th century, there were over 500 coffeehouses in Istanbul [8].

Coffee reached Europe in the 17th century as a result of the Ottoman Empire’s invasions. The first European coffeehouse was established in Oxford around 1650, and by the end of the 17th century, others had opened in Marseille, Paris, Amsterdam, Vienna, and Hamburg. Coffee quickly spread throughout Europe, becoming an integral part of social and cultural life [7,8]. By the 18th century, coffee production was still confined to the Yemen and Ethiopia region; however, its immense popularity led to its cultivation in other parts of the world, such as the Caribbean, Brazil, and Colombia, contributing to the development of the global coffee market [6,8].

Until coffee spread throughout the Muslim world and conquered Europe, it was always regarded as an elixir, a special source of energy that improved health, stimulated intellectual development, and treated various ailments [8,10].

Over centuries, coffee became integral to global cultures, sparking scientific curiosity. The 16th–17th century scientific revolution fueled interest in its chemistry, and by the 19th century, research intensified, especially in France, Germany, and Britain. A key breakthrough came in 1819 when German chemist Friedlieb Runge discovered caffeine, explaining coffee’s effects and its pharmacological significance [10].

In 1895, German chemist Hermann Emil Fischer (1852–1919) was the first to synthesize caffeine, which was a significant scientific breakthrough. This achievement contributed to Fischer winning the Nobel Prize in 1902 and enabled a better understanding of the mechanism of caffeine’s action [11]. Along with the discovery of caffeine, scientists also identified thousands of other chemical substances present in coffee, including carbohydrates, lipids, nitrogen compounds, vitamins, minerals, alkaloids, and phenolic compounds. These components not only give coffee its characteristic flavor but also have a positive impact on human health and mental function.

## 2. Composition of Coffee

The chemical composition of coffee is highly complex and includes a wide range of compounds that determine both its sensory qualities and its health effects. Among the most important components of coffee are alkaloids, such as caffeine, which is responsible for its stimulating effect, and polyphenols, including chlorogenic acid (CGA), which has antioxidant properties [12]. Coffee beans also contain carbohydrates, lipids, and numerous volatile compounds that shape the rich aroma and flavor of the brew, depending on the roasting process. Additionally, coffee is a source of minerals such as magnesium and potassium, which support bodily functions. During the roasting of coffee beans, chemical reactions such as the Maillard reaction, caramelization, Strecker degradation, polyphenol degradation, carbohydrate polymerization, and pyrolysis occur, affecting the color, taste, and aroma of coffee [13].

It is this complexity of coffee’s chemical composition that makes it not only a popular element of the daily diet but also a subject of interest for researchers who analyze its health and sensory properties.

### 2.1. Alkaloids

Caffeine, one of the main alkaloids present in coffee, has a chemical structure based on a purine ring with partial charges that promote dimerization in solution [14]. It is a heterocyclic compound belonging to the methylxanthine group, consisting of two nitrogenous rings and three methyl groups attached to nitrogen atoms. Its chemical formula is C_8_H_10_N_4_O_2_. Caffeine is highly soluble in water, with a solubility of approximately 21.7 mg/mL at 25 °C [15], which facilitates its extraction during coffee brewing. Importantly, caffeine exhibits temperature-dependent solubility, increasing significantly with rising temperature. This property is critical in coffee preparation, as higher brewing temperatures enhance caffeine extraction, directly impacting the caffeine content in coffee beverages [12,16]. It has a stimulating effect because, as an adenosine receptor antagonist in the brain, it inhibits the action of adenosine, a neurotransmitter responsible for the feeling of drowsiness. Caffeine is also lipophilic, allowing it to cross the blood–brain barrier, contributing to its rapid impact on the nervous system [17]. The physicochemical properties of caffeine, such as its melting point (235–238 °C) and low toxicity, make it safe for consumption in moderate amounts [18]. In addition to caffeine, coffee contains trigonelline, another alkaloid that undergoes degradation during roasting, contributing to flavor development. The quantity of caffeine and trigonelline in *C. arabica* berries varies depending on factors such as cultivar and processing methods [19]. On average, raw *C. arabica* beans contain 0.9–1.2% caffeine and 1–1.2% trigonelline by dry weight. In brewed coffee, the concentration of these alkaloids depends on brewing parameters such as grind size, water temperature, and extraction time [20]. Trigonelline is particularly sensitive to thermal degradation, converting into nicotinic acid and forming key flavor compounds during roasting [19]. Caffeine exhibits linear pharmacokinetics, where increasing the dose leads to a proportional rise in blood concentration. Approximately 20% is absorbed in the stomach, while the majority (80%) is taken up in the small intestine, which indicates its high bioaccessibility. Once in circulation, 10–30% binds reversibly to plasma proteins such as albumin, with the remainder quickly dispersing throughout the body [21]. Although it does not accumulate in tissues, high concentrations are found in the brain [22]. Gastrosimulation experiments show that caffeine is relatively stable under gastrointestinal conditions, and its release depends mainly on the type of coffee and the degree of processing. A study comparing the pharmacokinetics of a single dose of caffeine from a coffee enema (107.2 ± 2.2 mg) versus oral coffee consumption (96.3 ± 1.3 mg) in healthy men found that caffeine’s relative bioavailability from the enema was about 3.5 times lower than from oral intake [23].

### 2.2. Diterpenes

Diterpenes, such as cafestol and kahweol, are lipid-soluble compounds found in coffee oil. These bioactive compounds exhibit potential health effects, including anti-inflammatory and cholesterol-modulating properties. However, their concentration in coffee depends on the brewing method, with unfiltered coffee (such as French press and espresso) containing higher levels than filtered coffee [24].

### 2.3. Polyphenols

Coffee is one of the main sources of antioxidant polyphenols in the diet, with its total polyphenol content being higher than that of other beverages, including green tea. The average intake of polyphenols from coffee is about 426 mg per day. The most common polyphenols in coffee include CGA and its derivatives, which significantly contribute to coffee’s antioxidant properties [25]. The roasting temperature of coffee beans significantly affects their polyphenol profile through the Maillard reaction, which simultaneously gives coffee its characteristic aroma and flavor [13].

### 2.4. Chlorogenic Acid

CGA is a phenolic compound formed from caffeic acid and quinic acid, produced via the shikimic acid pathway in plants. It occurs in various isomeric forms, mainly as mono-caffeoylquinic and di-caffeoylquinic acids [26]. The structure of CGA consists of a quinic acid core esterified with caffeic acid, which contributes to its biological activity, including antioxidant and antibacterial properties [17]. The concentration of CGA in *C. arabica* berries varies between 5.5–8% [20] of the dry weight, with the most abundant derivatives including neochlorogenic acid, cryptochlorogenic acid, and dicaffeoylquinic acid [27]. In the twenty-four analyzed coffee brews, the predominant isomer was 3-caffeoylquinic acid, comprising approximately 50% of the total caffeoylquinic acids. It was followed by 5-caffeoylquinic acid and 4-caffeoylquinic acid, each accounting for about 24–36%. The total concentration of caffeoylquinic acids ranged from 45.79 to 1662.01 mg/L, depending on the type of coffee [28].

During coffee preparation, the extractability and stability of CGA are influenced by factors such as roasting and brewing temperature. Roasting leads to partial degradation of CGA, converting them into derivatives with altered bioactivity. The brewing process also affects CGA retention, with higher temperatures generally increasing extraction but also promoting hydrolysis and oxidation [29].

The antioxidant properties of CGA result from its ability to neutralize free radicals, preventing oxidative stress in the body, which is a factor that accelerates aging processes and the development of chronic diseases [30]. CGA exhibits a multifaceted antioxidant mechanism. Its polyhydroxyl structure enables the direct scavenging and neutralization of free radicals, protecting cells from oxidative stress. Furthermore, CGA activates signaling pathways linked to antioxidant defenses, influencing the regulation of genes responsible for these processes and boosting the body’s ability to neutralize reactive oxygen species (ROS). CGA was found to reduce the expression of Keap1, a key regulator of the cellular oxidative stress response. This reduction activates the nuclear factor erythroid 2-related factor 2 (Nrf2) signaling pathway, which is highly sensitive to OS [26].

Additionally, CGA modulates the activity of oxidase enzymes and associated endogenous proteins, further supporting antioxidant mechanisms and enhancing cellular protection against free radical-induced damage [26]. CGA modulates the expression of proteins and genes involved in the inflammatory response, thereby mitigating various inflammation-induced damages to the body. This is primarily achieved through the activation of the Nrf2/HO-1 pathway and the inhibition of the nuclear factor kappa-light-chain-enhancer of activated B cells pathway [31,32]. Furthermore, CGA has been shown to enhance neuronal cell viability, inhibit the transformation of the autophagosome marker, and reverse the decreased activity of some cellular pathways [33]. These effects contribute to its neuroprotective properties and potential therapeutic benefits in neurodegenerative conditions.

### 2.5. Carbohydrates

Raw coffee beans contain a significant amount of polysaccharides, including galactomannans, which constitute up to 50% of the dry weight of the beans, as well as arabinogalactans and cellulose, which are structural components of the cell walls of the beans [34]. During the roasting process, complex chemical reactions occur, such as caramelization and Maillard reactions, involving both simple and complex sugars. Caramelization, a non-enzymatic browning reaction, occurs at high temperatures and leads to the formation of caramelized sugars, which contribute to the characteristic color and sweetness of roasted coffee [35]. The Maillard reaction, on the other hand, is a complex reaction between amino acids and reducing sugars that produces melanoidins, compounds responsible for coffee’s deep brown color and roasted aroma [36]. These reactions also generate key flavor and aroma compounds such as pyrazines and furans, which define the sensory profile of coffee [37].

After this stage, 20–40% of the sugars degrade, primarily galactose and mannose, due to their high thermal sensitivity. The reactions occurring during coffee roasting are responsible for the formation of aromatic compounds and pigments that give coffee its characteristic taste, color, and aroma [38].

In brewed coffee, carbohydrates are primarily present as soluble simple sugars, including glucose and fructose, along with oligosaccharides. Additionally, coffee contains small amounts of dietary fiber, which, though not fully soluble in water, may contribute to digestive health [39]. Brewed coffee contains soluble dietary fiber in amounts ranging from 0.47 to 0.75 g per 100 mL, depending on the type of coffee [40].

### 2.6. Lipids

The lipid content in green coffee beans averages between 7 and 17%. Coffee oil consists mainly of triglycerides with fatty acids in proportions similar to those found in common edible vegetable oils [41]. Most of the acids present in coffee are triglycerides (75–85%), diterpene esters (≤20%), sterols (3.2%), tocopherols (0.05%), phosphatides (0.3%), and tryptamine derivatives (≤1%). Green coffee beans contain free fatty acids, such as linoleic and palmitic acids, as well as secondary acids, such as myristic, palmitoleic, eicosanoic, behenic, and arachidic acids. These acids occur in three forms: approximately 49.4–59.2% are saturated fatty acids, 4.3–9.69% are monounsaturated fatty acids, and polyunsaturated fatty acids constitute 29.5–39.2% [42,43,44].

### 2.7. Volatile Compounds

Coffee contains a diverse range of volatile compounds, including aldehydes, ketones, alcohols, esters, pyrazines, furans, acids, nitrogen compounds, and volatile phenolic compounds. These substances form the foundation of coffee’s unique aroma profile—arguably its most distinctive characteristic. Their concentrations can vary significantly depending on the methods used to process the beans [45]. These compounds are highly sensitive to changes in temperature and roasting time, meaning that even small differences in bean processing can lead to significant changes in the aroma and flavor of the brew. For example, aldehydes such as furfural contribute to caramel and sweet notes, while phenols contribute to smoky and burnt notes. Esters, on the other hand, give coffee fruity and floral notes [46]. It is also worth noting that the intensity and persistence of volatile compounds in the finished brew depend on the method of preparation [11].

The complexity and diversity of volatile compounds make coffee’s aroma one of the world’s most distinctive and cherished qualities, captivating consumers with its rich sensory profile.

### 2.8. Minerals and Trace Elements

Among the most important minerals present in coffee are potassium, magnesium, calcium, and phosphorus. Potassium, which is present in the largest amounts (about 40%), plays a key role in regulating the body’s water–electrolyte balance and supports the functioning of muscles and the nervous system [47]. Potassium is an element that freely penetrates coffee brews and is highly soluble [48]. For ground and roasted coffees, the extraction efficiency of potassium is high, ranging from 72.9–88.6% [47].

Magnesium is essential for the proper course of many metabolic processes, including protein synthesis and enzyme activity [49]. According to the European Food Safety Authority, the daily requirement for this element is 350 mg for men and 300 mg for women [50]. Due to insufficient consumption of fruits, vegetables, and whole grain products, an average of 48% of the U.S. population of all ages suffers from magnesium deficiency, and a cup or two of coffee can help slightly reduce this gap [51]. Considering the average content of this element per 100 mL of the beverage, the content in a cup (150 mL) ranges from 3.22 mg to 22.38 mg, covering 1.1–7.5% of the daily magnesium requirement for women and 0.9–6.4% for men, making it a small but significant contribution to the daily intake of this mineral [47].

Coffee contains small amounts of calcium, which is essential for bone and dental health as well as playing an important role in nerve impulse conduction and muscle contractions [47]. Considering the daily calcium requirement, coffee would cover 0.3 to 0.7% of the daily requirement for women and 0.2 to 0.6% of the daily requirement for men [47]. Nevertheless, a study of over 400,000 people showed a correlation between coffee consumption and a reduction in osteoporosis and fractures [52].

Coffee also contains trace elements such as iron, zinc, copper, and manganese, which support the proper functioning of the body [53]. While the minerals and trace elements in a single cup of coffee are insufficient to meet daily nutritional requirements, regular coffee consumption can serve as a supplementary source of these nutrients, contributing to the body’s overall mineral balance. Furthermore, coffee is known to enhance metabolism, offering potential benefits for those managing their body weight.

During the brewing process, various chemical compounds from coffee beans transfer into the beverage, including those that influence its taste, aroma, and health properties. CGA partially breaks down during roasting [54]. Caffeic acid and ferulic acid are formed from the breakdown of CGA and are found, for example, in filtered coffee and espresso. Quinic acid is also a product of CGA degradation and is responsible for the bitterness in coffee [55]. Citric, malic, lactic, and acetic acids give coffee its acidity and fresh taste [55]. Caffeine completely transfers into the brew and has a stimulating effect. Trigonelline partially decomposes during roasting, but its remnants contribute to the aroma of coffee [56]. Furthermore, melanoidins are formed in the Maillard reaction and are present in roasted coffee [57].

Moreover, oligosaccharides and monosaccharides partially transfer into the beverage and influence its taste [58]. An example is naturally sweeter coffees, such as certain Arabica varieties. Diterpenes, such as cafestol and kahweol, are found in coffee oils. They remain in espresso and coffee brewed using the French press method but are partially filtered out in pour-over methods [24]. Finally, furans, pyrazines, aldehydes, ketones, and esters contribute to the aroma of coffee. These compounds are mainly released during brewing and give the beverage its characteristic scent [59].

## 3. Materials and Methods

A comprehensive literature search was carried out using the PubMed, Scopus, and Web of Science databases. This review focused on articles published in English between 2000 and 2025. Each identified study underwent a three-stage evaluation process, including title screening, abstract review, and full-text analysis, to determine its relevance. The search strategy was based on key terms such as “Coffee and oxidative stress” and “Coffee and anti-aging”. Studies were excluded from the review if they did not meet specific criteria. Articles that were not published in peer-reviewed journals were omitted to ensure the reliability of the findings. Research that did not explicitly address the link between coffee and oxidative stress or anti-aging was also not considered. Only publications written in English were included in the review.

## 4. Coffee and Oxidative Stress

**Figure 1:**
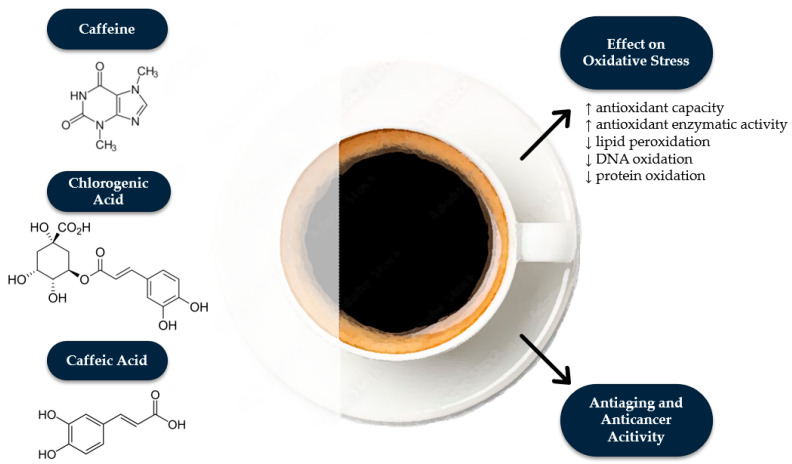
The main active compounds in coffee and their positive effects.

OS arises when there is a disruption between the generation of ROS and the body’s capacity to counteract them using defense mechanisms, such as antioxidant enzymes or chemical compounds with antioxidant properties [60]. OS plays a crucial role in the onset and advancement of diseases, including atherosclerosis and neurodegenerative disorders like Alzheimer’s disease [61]. Moreover, ROS damage biomolecules such as proteins, lipids, and nucleic acids through direct oxidation or by initiating chain reactions. As a result, they cause protein denaturation, lipid peroxidation, and DNA mutations, which can disrupt cellular functions and contribute to disease development [62]. Drinking coffee rich in antioxidants may therefore offer health benefits by helping protect cells from the harmful effects of ROS (Figure 1).

Epidemiological research indicates that regular coffee intake is correlated with a lower likelihood of developing diseases related to OS [2,63,64]. Emerging evidence suggests that moderate coffee consumption (1–4 cups per day) may be associated with a reduced risk of neurodegenerative disorders, including Parkinson’s and Alzheimer’s diseases [65,66,67,68]. However, some studies have reported no significant association between coffee intake and the risk of Alzheimer’s disease [69].

Interestingly, among hypertensive individuals, a lower incidence of dementia has been observed in those consuming 0.5–1 cup of coffee per day compared to the non-hypertensive population [70]. Additionally, a daily intake of 2–3 cups has been linked to the lowest hazard ratio for both stroke and dementia [71]. A study conducted in an older Japanese population further reported that consuming three or more cups of coffee per day was associated with a 50% reduction in dementia risk [72].

With regard to cancer risk, epidemiological studies indicate an inverse relationship between coffee consumption and the incidence of certain malignancies. A meta-analysis demonstrated that increased coffee intake is associated with a lower risk of prostate cancer [73], while another analysis suggested that coffee consumption may be linked to a reduced risk of head and neck cancers [74]. However, the relationship between coffee intake and gastric cancer remains inconclusive. While no significant nonlinear association has been identified [75], excessive consumption exceeding 6.5 cups per day has been correlated with an increased risk of gastric cancer in the U.S. population [76].

Beyond neuroprotection and cancer prevention, moderate coffee consumption has been linked to additional health benefits. A daily intake of 1–2 cups has been associated with a reduced incidence of osteoporosis [77], and habitual moderate coffee consumption appears to lower the risk of developing cataracts [78]. Furthermore, consuming more than three cups per day has been shown to significantly reduce the prevalence of nonalcoholic fatty liver disease [79].

The potential health-promoting properties of coffee are largely attributed to its high content of bioactive compounds, particularly chlorogenic acids, which exhibit potent antioxidant and anti-inflammatory effects [80]. These compounds may play a crucial role in mitigating oxidative stress, a key driver in the pathogenesis of various chronic diseases.

Nevertheless, while epidemiological data provide compelling associations between coffee consumption and reduced disease risk, these findings do not establish a direct causal relationship. Confounding variables, heterogeneity in study designs, and population-specific differences may influence observed outcomes. Consequently, while the current evidence is promising, further research—particularly well-controlled clinical trials—is necessary to elucidate the precise role of coffee in mitigating oxidative stress-related diseases.

While coffee consumption offers numerous health benefits, direct caffeine administration has also been shown to positively affect OS markers. In a study involving healthy male participants, a one-week regimen of pure caffeine at a dose of 5 mg/kg body weight per day, split into two daily doses, resulted in reduced lipid peroxidation and increased levels of reduced glutathione and total antioxidant capacity (TAC) [81]. Coffee may affect the nuclear factor kappa-light-chain-enhancer of activated B cell signaling pathways, which is crucial in inflammation and OS [82,83]. Activation of this pathway in response to OS can trigger the production of proinflammatory cytokines, amplifying the inflammatory response. However, compounds found in coffee, particularly polyphenols, can stimulate the Nrf2 pathway, which governs the expression of genes involved in antioxidant defense and detoxification [84].

CGA, a prominent polyphenol in coffee, has been shown to activate the Nrf2 pathway, leading to the upregulation of antioxidant response element (ARE)-mediated expression of phase II detoxifying enzymes. This activation enhances the body’s antioxidant defenses and supports detoxification processes [85]. Moreover, cafestol and kahweol, two diterpenes found in coffee, have demonstrated the ability to modulate the Nrf2/ARE pathway. Their activation of Nrf2 leads to increased expression of phase II detoxifying enzymes, such as glutathione S-transferases and NAD(P)H:quinone oxidoreductase 1, which play crucial roles in detoxifying ROS and other harmful compounds [24]. Caffeic acid, another compound present in coffee, has been reported to exhibit antioxidant properties and the potential to activate the Nrf2 pathway, thereby contributing to the induction of phase II detoxifying enzymes [86,87].

In addition, coffee has been shown to counteract the activation of signaling pathways associated with pro-inflammatory molecules such as tumor necrosis factor-α and interleukin-6 [88]. By modulating these pathways, coffee can contribute to reducing inflammation and OS in the body. Numerous studies have documented the impact of coffee consumption on OS markers in both in vivo and in vitro models.

### 4.1. The Effect of Coffee on Antioxidant Capacity

Studies examining the effect of coffee intake on plasma/serum antioxidant capacity are not conclusive and indicate an increase in TAC after coffee consumption [89,90,91] or no changes in the level of this parameter [92,93,94].

Colombian Arabica coffee, rich in CGA and low in diterpenes and caffeine, showed a positive acute effect on plasma TAC in healthy adults. However, these effects diminished and were no longer observed following long-term consumption [89]. Ninety minutes after a single consumption of 200 mL of coffee, plasma TAC increased by 2.6–7.6%, depending on the estimation methods, compared to baseline levels in ten studied adults [95]. Additionally, a 4-week intake of 150 mL/day of paper-filtered coffee, either medium light roast or medium roast, increased total antioxidant status by approximately 21% and 26%, respectively. However, a significant increase in oxygen radical absorbance capacity was observed only with medium light roast paper-filtered coffee [96]. Moreover, a study by Natella et al. [90] proved that consumption of 200 mL of coffee led to an increase in the plasma TAC in humans, accompanied by a significant rise in plasma uric acid levels. On the other hand, there were no observed positive effects in terms of improving overall TAC after using a coffee enema or consuming ready-to-drink coffee orally in acute and long-term interventions [92]. Additionally, acute coffee consumption after a high-fat milkshake did not affect postprandial OS, as measured by TAC level [93]. Generally, Robusta coffee showed higher TAC than Arabica in in vitro studies [97]. Furthermore, a recent study found that different varieties of green Yemeni coffee beans exhibit different antioxidant properties [98]. Interestingly, the coffee pulp, a by-product of the coffee industry, also exhibited increased TAC and demonstrated ROS scavenging activity even after undergoing in vitro digestion [99]. The variability in results may be due to differences in the intervention duration, the type and quantity of coffee consumed, the composition of bioactive compounds, and the methods used to evaluate antioxidant capacity [100].

The polyphenol and caffeine content varies based on coffee bean type, roasting degree, and brewing method [101,102]. Research indicates that both acute and chronic coffee consumption can enhance blood antioxidant capacity [90,93]. Higher consumption levels may lead to more pronounced effects due to the cumulative impact of bioactive compounds, whereas low or occasional intake may not produce measurable changes in TAC. TAC assessment methods (e.g., FRAP, ORAC, DPPH) differ in specificity, affecting comparability across studies [103]. Moreover, CGAs undergo metabolic transformations during digestion, impacting their bioavailability and subsequent effects on serum TAC [104]. In detail, upon ingestion, CGAs are hydrolyzed by esterases in the small intestine, releasing caffeic and quinic acids. These metabolites are then absorbed, conjugated in the liver, and further metabolized by the colonic microbiota, producing various derivatives that enter systemic circulation [105]. The bioavailability of these compounds is relatively low, with studies indicating that approximately one-third of ingested CGAs are absorbed in the gastrointestinal tract. This limited bioavailability affects their efficacy in enhancing serum TAC, as the antioxidant activity of CGAs and their metabolites depends on their concentration and form in the bloodstream [106,107].

### 4.2. The Effect of Coffee on Antioxidant Enzymatic Activity

Antioxidant enzymes, including superoxide dismutase (SOD), catalase (CAT), and glutathione peroxidase (GPx), are essential for protecting cells from oxidative damage by facilitating the breakdown of ROS and preserving redox balance. A significant effect of coffee intake on the activity of antioxidant enzymes such as SOD, GPx, and CAT was noted in a study among healthy volunteers by Corrêa et al. [96]. CAT and SOD activities were significantly higher in the coffee consumption group of rats than the control group in a study by Choi et al. [108]. Activity of GPx was only marginally increased after consumption of 400 mL paper-filtered and 200 ml metal-filtered coffee per day, while a significant enhancement in SOD activity was observed [109]. Furthermore, consumption of dark roast coffee most effectively decreased SOD and GPx activity in erythrocytes of healthy volunteers [110]. On the other hand, activities of SOD and GPx in lymphocytes were at the same level after consumption of 800 mL paper-filtered coffee for 5 days [94]. Consumption of a brew made from a special Arabica coffee variety led to an increase in glutathione reductase activity [111]. Moreover, a Turkish study on rats conducted by Demir et al. [112] found that coffee intake resulted in a decrease in glutathione S-transferase activity but did not have an effect on SOD activity in the kidney tissue. Increased activity of antioxidant enzymes may contribute to protection against diseases related to OS.

### 4.3. The Effect of Coffee on Lipid Peroxidation

Coffee, due to its antioxidant content, may positively influence the reduction of lipid peroxidation by lowering levels of markers, including malondialdehyde (MDA) and F2-isoprostanes, which are widely recognized as indicators of lipid peroxidation. Coffee consumption has been associated with a reduction in oxidative stress and lipid peroxidation, primarily due to its high content of bioactive compounds, including CGA, caffeic acid, and kahweol. These compounds exert antioxidant effects by modulating endogenous defense mechanisms, such as activating the Nrf2 pathway, which leads to the upregulation of detoxifying enzymes, including GPx, SOD, and CAT. For instance, caffeine has been shown to scavenge ROS, particularly hydroxyl radicals, thereby preventing oxidative damage to lipids [113]. This activity contributes to the reduction of lipid peroxidation products, such as MDA, a byproduct of lipid peroxidation, where ROS initiate oxidative degradation of polyunsaturated fatty acids. Studies indicate that coffee and its bioactive compounds influence the regulation of transcription factors and enzymes involved in lipid metabolism processes, including lipogenesis, lipid uptake and transport, β-oxidation of fatty acids, and lipolysis [114,115,116].

The interaction between coffee-derived antioxidants and lipid metabolism also plays a role in maintaining cell membrane integrity and vascular function, which may help explain the observed correlation between coffee consumption and the incidence of oxidative stress-related diseases. However, research in this area in humans remains inconclusive.

No significant changes in lipid peroxidation biomarkers were observed following the consumption of medium light roast or medium roast paper-filtered coffee in healthy volunteers [96]. On the other hand, a recent study by Martini et al. [117] reported a significant increase in total F2-isoprostanes (5-series) only after consuming three cups of espresso coffee per day. Furthermore, levels of individual oxylipins remained unchanged regardless of the consumption of the one or three espresso cups a day [117]. Urinary 8-isoprostaglandin F2α decreased by 15.3% in participants who consumed instant coffee co-extracted from green and roasted beans compared to the control group that consumed water [91]. Similarly, consuming 8 cups of coffee per day for 4 weeks, following a 4-week restrictive diet and 4 weeks of consuming 4 cups of coffee per day, led to a decrease in serum 8-isoprostane levels in coffee consumers [118]. Moreover, the resistance of low-density lipoprotein to oxidative modification was significantly enhanced after consuming 1 cup of filtered coffee in healthy volunteers [119]. Nevertheless, plasma MDA and urinary 8-isoprostaglandine F2α levels were unchanged after consumption of 800 mL paper-filtered coffee for 5 days in a study by Mišík et al. [94]. Interestingly, rats from the coffee intake group exhibited higher serum MDA levels compared to the control group [108]. Furthermore, studies on healthy volunteers consuming red meat have shown that drinking coffee with a meal reduces the postprandial absorption of MDA into circulating plasma [120]. It appears that the storage conditions of green coffee beans before roasting directly impact the beans’ lipid oxidation [121]. The results of studies are varied, and the effects may depend on several factors, including the type of coffee, the amount consumed, and individual differences in health and diet. Further research is needed to more precisely determine how coffee affects lipid peroxidation.

### 4.4. The Effect of Coffee on DNA Oxidation

The impact of coffee on oxidative DNA damage is complex and may depend on various factors such as the chemical composition of the coffee, the amount consumed, the duration of consumption, and individual characteristics of the study subjects. Research on this topic has yielded mixed results. Several studies indicate that regular coffee consumption may lower levels of biomarkers associated with oxidative DNA damage.

Three cups of espresso coffee a day significantly decreased DNA strand breakage after one month of treatment, and it was more effective in reducing DNA strand breakage than consuming 1 cup of espresso a day. Consuming 400 mL of paper-filtered coffee and 200 mL of metal-filtered coffee daily for five days resulted in a reduction in DNA damage linked to ROS and the heterocyclic aromatic amine 3-amino-1-methyl-5H-pyrido [4,3-b]indole-acetate by 17% and 35%, respectively [109]. Habitual coffee intake has been linked to a reduction in baseline DNA strand breakage [122,123]. Further, protective effects of coffee against spontaneous DNA damage induction was also confirmed in human and animal studies [124,125]. Furthermore, after consuming 800 mL of coffee daily for 5 days, the degree of DNA migration resulting from the formation of oxidized purines decreased by 12.3% [94]. Urinary 8-hydroxydeoxyguanosine level showed a tendency to decline with coffee consumption in women, with those drinking 2–3 cups per day exhibiting the lowest average levels of urinary DNA damage [126]. Nevertheless, no effects of one or three espresso cups were observed on H_2_O_2_-induced DNA damage or formamidopyrimidine DNA glycosylase-sensitive sites [117]. Moreover, no effects were reported in plasma DNA oxidation catabolites after any of the two coffee interventions [117]. Likewise, a placebo-controlled intervention trial conducted on 160 healthy human subjects revealed that consuming up to 5 cups of coffee per day had no detectable effect on DNA strand breaks and oxidized bases [127]. Surprisingly, a 130-day coffee diet resulted in a significant rise in the excretion of urinary 8-hydroxydeoxyguanosine in rats, a major marker of oxidative DNA damage [128]. Coffee is a free radical scavenger of the Yap1, a transcription factor accumulated in the *Saccharomyces cerevisiae* cell nucleus during OS, fusion protein. Moreover, coffee protects cells by preventing DNA double-strand breaks [97]. While coffee contains compounds with potentially protective effects against oxidative DNA damage, the scientific evidence is not conclusive.

### 4.5. The Effect of Coffee on Protein Oxidation

Clinical research on the impact of coffee consumption on oxidative protein damage can be mixed. Some studies indicate a beneficial effect of coffee in reducing oxidative protein damage, while others show no significant effects. These differences may arise from variations in study methodologies, coffee brewing methods, amounts of coffee consumed, and individual differences in health and diet among participants.

Five-day consumption of instant coffee co-extracted from green and roasted beans for 4 weeks resulted in a 16.1% reduction in 3-nitrotyrosine (3-NT) concentration, a stable marker of post-translational protein modification [91]. However, 3-NT concentration was similar before and after consumption of 800 mL paper-filtered coffee for 5 days [94]. Likewise, drinking 8 cups of coffee per day for 4 weeks had no effect on serum 3-NT levels in coffee consumers in an experiment conducted by Kempf et al. [118]. Discrepancies between study results may be due to different types of coffee and their brewing methods. In addition, oral administration of caffeine at two high doses (30 mg/kg and 100 mg/kg) led to a reduction in advanced oxidation protein products in rat kidneys [112]. Moreover, a recent study by Tatar and Koç [129] proved that consumption of Turkish coffee was negatively correlated with glycated hemoglobin percentage in participants with type 2 diabetes mellitus. This effect is due to the reduction of oxidative cellular damage and the improvement of overall cellular health.

In summary, coffee, thanks to its rich antioxidant content, may effectively reduce OS, protecting cells from the damage caused by ROS and modulating signaling pathways responsible for the antioxidant response. Nevertheless, despite promising data, further research is needed in the form of both in vitro and in vivo studies.

## 5. Coffee Plays a Crucial Role in Aging and Healthspan

Aging, a universal and inevitable process, has long been a subject of intense scientific scrutiny, with researchers striving to unravel the complex mechanisms underlying this intricate phenomenon. In a recent article, Suresh Rattan [130] sought to consolidate the existing knowledge and highlight the gaps in the field of biogerontology, the study of the biological aspects of aging. Aging is a multifaceted process that encompasses a wide range of physiological, biochemical, and molecular changes. The reversible nature of epigenetic information, as demonstrated by recent findings, has opened up exciting avenues for therapeutic interventions in aging and age-associated diseases, including cancer [131]. Aging increases the susceptibility to various disorders, such as metabolic, cardiovascular, and neurodegenerative diseases, underscoring the importance of understanding its underlying mechanisms [131].

Longevity is a dream cherished by many people, but it is equally important for this life to be not only long but also healthy and fulfilling. There is a significant difference between simply achieving longevity and living a healthy and fulfilling life during those years. Longevity means simply reaching an advanced age. It can be the result of genetics, luck, and appropriate living conditions. However, merely reaching an old age does not guarantee that this time will be spent in health and wellbeing. In contrast, a healthy long life refers to spending those additional years in good health, maintaining physical and mental fitness. Healthy longevity is often the result of a healthy lifestyle, which includes a proper diet, regular physical activity, avoiding harmful habits, and taking care of mental and social health [132]. Today, while people are living longer, many are also contending with multiple chronic diseases that often severely impact their quality of life [133]. In recent decades, significant advancements in medical care and public health measures have led to a remarkable increase in human lifespan across many regions of the world [134,135]. However, this remarkable achievement has been accompanied by a concerning trend—the rise of chronic diseases that often severely impact the quality of life for the elderly population. The last 20 years appear to have been pivotal in understanding the biology of aging. The main factors closely associated with aging are identified as adaptation to stress, macromolecular damage, metabolism, epigenetics, inflammation, and proteostasis [136]. Scientific studies suggest that coffee has beneficial effects on health and survival [137]. The interest in the properties of coffee seems justified given that it is the second most consumed beverage after water [138]. In aging research, a variety of model systems are utilized, which serve as key tools in biochemical, genetic, and epigenetic studies. These models include the nematode *Caenorhabditis elegans*, the fruit fly *Drosophila melanogaster*, and the yeast *S. cerevisiae*. Among vertebrates, the mouse and rat play a primary role. The potential impact of coffee consumption on aging has been explored in various model organisms, including yeast, nematodes, and rodents. In addition to coffee, its components, such as caffeine and CGA, play a significant role in aging. Recent studies using the nematode *C. elegans* have shown that caffeine increases its lifespan, delays larval development, and reduces reproduction and body length. Interestingly, this phenotype is partially dependent on adenosine signaling [139]. Subsequent reports showed that a 10 μg/mL concentration of caffeine extended the lifespan of *C. elegans* without affecting food intake and reproduction. This effect was dependent on the insulin-like growth factor 1 (IGF-1-like) pathway. Additionally, four caffeine analogs were discovered to extend lifespan: 1-methylxanthine, 7-methylxanthine, 1,3-dimethylxanthine, and 1,7-dimethylxanthine [140]. These caffeine analogs are present in trace amounts in coffee or have not been discovered in plants [141,142,143]. Importantly, all these analogs are metabolized by our organism, with the primary enzymes responsible for caffeine catabolism being the products of the *CYP1A2* and *CYP2E1* genes [144].

Low concentrations of caffeine also have a protective role in aging-related disorders in the nematode model [145]. To investigate the impact of caffeine on the longevity of nematodes, *C. elegans* were treated with 1 mM and 5 mM concentrations of caffeine. Both concentrations extended the lifespan of the worms, with the 5 mM caffeine treatment showing a more pronounced effect, increasing the lifespan of the N2 strain by 22.22%. The results indicate that, compared to the untreated control, the lifespan of the DAF-16 and SKN-1 mutant strains was also extended when exposed to caffeine. This suggests that the lifespan-extending effects of caffeine are not strictly dependent on the DAF-16 and SKN-1 pathways [145].

Long-term caffeine intake in *C. elegans* exerts a protective effect on intestinal aging through the regulation of vitellogenesis [146]. Recent reports show that kahweol, a major diterpene in coffee, extends the lifespan of *C. elegans* via an insulin/insulin-like growth factor pathway [147]. The results demonstrated a significant extension in the lifespan of *C. elegans* starting at a concentration of 10 μM of kahweol. The maximum lifespan extension was observed at a 25 μM treatment, resulting in an approximate 28% increase in mean lifespan compared to the control [147].

Previous studies have also demonstrated that a 50 mM concentration of CGA could prolong the adult mean lifespan of N2 *C. elegans* by up to 20.1%. Additionally, CGA was shown to slow down the deterioration of body movement associated with aging and enhance resistance to stress [148]. Moreover, recent studies have demonstrated that CGA has significant effects on aging in the model organism *C. elegans*. CGA has been shown to extend the lifespan of *C. elegans*, delay age-related declines in body movement, and enhance stress resistance. The underlying mechanisms involve the activation of FOXO transcription factors, specifically DAF-16, HSF-1, SKN-1, and HIF-1, but not SIR-2.1 [148,149].

Furthermore, recent studies confirm that coffee positively affects the survival of *S. cerevisiae* [148,149]. Previously, Czachor et al. [97] provided strong evidence that coffee compounds, particularly flavonoids, are responsible for longevity in non-mitotic budding yeast cells.

The analysis of postmitotic cell aging was conducted using mutants lacking antioxidant protection, *sod1*Δ and *sod2*Δ, as well as a strain sensitive to genotoxic stress, *rad52*Δ. Coffee infusions from both Robusta and Arabica were used as the material. The studies indicated that Arabica coffee has a stronger effect on delaying aging in yeast cells. It was concluded that coffee infusions extend the chronological lifespan of yeast cells by protecting them against ROS and double-strand DNA breaks [97].

Numerous studies have reported the positive effects of coffee consumption on various aspects of aging. Coffee, rich in bioactive compounds such as caffeine and polyphenols, has been shown to influence energy metabolism and longevity in aged mice. Furthermore, habitual coffee intake has been associated with reduced all-cause mortality and a decreased risk of cardiovascular and cerebrovascular diseases in multiple population groups [150]. Delving deeper into the mechanisms behind coffee’s anti-aging effects, research has highlighted the role of caffeine in inhibiting the mammalian target of rapamycin complex 1, a key regulator of cellular aging. Additionally, the polyphenol content of coffee has been linked to improved energy production and decreased levels of the aging-related protein mammalian target of rapamycin (mTOR) in aged mice [150].

These compounds have been shown to possess anti-inflammatory, antioxidant, and neuroprotective properties, which may contribute to the observed improvements in lifespan, healthspan, and cognitive function in the studied model organisms [151,152,153,154]. Furthermore, emerging research indicates that coffee consumption may also modulate important cellular pathways involved in the regulation of aging, such as the insulin/IGF-1 signaling pathway and the AMP-activated protein kinase pathway [151,152,154,155]. Moreover, coffee has been shown to modulate key cellular pathways involved in the aging process, such as the mTOR pathway and the insulin-like growth factor signaling cascade [151,156]. These pathways play crucial roles in regulating cell growth, metabolism, and longevity, and their modulation by coffee components may contribute to the observed effects on aging.

While the insights gained from these model organism studies are valuable, it is important to note that translating these findings to human health and aging is not straightforward. Factors such as individual genetic predispositions, lifestyle factors, and the complex interplay of various bioactive compounds in the human body may influence the overall impact of coffee consumption on the aging process. Nonetheless, the evidence suggests that moderate coffee consumption may benefit aging, warranting further investigation in human clinical studies to elucidate the underlying mechanisms and potential implications for human health [151]. One area of interest is the potential impact of coffee on the aging adult population. Population studies suggest that coffee consumption is highly prevalent among the elderly, and coffee drinking provides exposure to a wide array of biologically active compounds and nutrients, including polyphenols, lipids, minerals, and notably, caffeine [157]. Interestingly, improvements in a variety of health outcomes, such as lower mortality, weight, cancer, diabetes, and patterns of markers of inflammation and insulin resistance, have been associated with coffee consumption, often exhibiting a dose-dependent relationship [157]. Emerging evidence suggests that coffee consumption may have a positive impact on neurocognitive function and neuroprotection, particularly in relation to conditions like Alzheimer’s disease. Population studies have consistently shown an inverse association between coffee consumption and all-cause mortality [158]. Coffee’s beneficial effects are often attributed to its rich composition of bioactive compounds, including polyphenols, lipids, minerals, and the renowned caffeine. Chronic coffee consumption has been associated with reduced risks for a variety of neurodegenerative diseases, such as Alzheimer’s and Parkinson’s, as well as improvements in cognitive performance and executive function [151,152]. The results indicate that coffee causes a short-term increase in blood pressure [159]. However, this temporary rise in blood pressure has been shown not to affect the risk of hypertension or is weakly and inversely associated with the risk of hypertension [160,161]. Currently, patients with cardiovascular disease are advised to avoid coffee due to increased blood pressure [3,162]. Interestingly, coffee consumption is associated with a reduced risk of type 2 diabetes [163]. The relationship between coffee and cardiovascular diseases appears to be complex and not straightforward. However, as previously shown, moderate coffee consumption is linked to a reduced risk of heart failure. Additionally, coffee consumption is associated with a lower risk of mortality [164,165,166,167]. The reduced lifespan benefits from higher coffee intake are anticipated due to the well-documented adverse effects of excessive coffee consumption on adult behavior. These effects include increased anxiety, agitation, restlessness, headaches, insomnia, and gastric distress [168]. Such symptoms can contribute to the development of caffeinism syndrome [169]. Additionally, high coffee consumption has been linked to conditions such as sarcopenia, frailty, and falls in older adults [157]. Table 1 presents studies on the effects of coffee on aging and healthspan.

## 6. Conclusions and Future Directions

Coffee, due to its high antioxidant content, may play a significant role in protecting the body against oxidative stress by supporting ROS neutralization and reducing the risk of biomolecular damage. Additionally, its bioactive components, such as polyphenols and caffeine, show potential in slowing aging processes and improving healthspan. The promising data on coffee’s health benefits and anti-aging properties open new avenues for research. Future studies should focus on human clinical trials, as much of the existing data are derived from in vitro and animal studies. Comprehensive longitudinal studies in diverse human populations are needed to confirm the long-term health benefits of coffee and establish optimal consumption patterns. Moreover, given the high prevalence of coffee consumption among the elderly, studies examining its effects on age-related conditions such as neurodegenerative diseases, osteoporosis, and frailty are particularly important. Future research should also examine how coffee interacts with other dietary elements, medications, or lifestyle factors to influence health outcomes. Another aspect may be personalized nutrition, in which exploring individual differences in genetic predisposition, metabolism, and microbiome composition can help tailor coffee consumption recommendations for maximum health benefits.

Coffee contains numerous antioxidants, yet the precise mechanisms by which these compounds influence aging remain only partially elucidated. Consequently, further research is imperative to clarify these molecular pathways. Additionally, while the anti-inflammatory effects of coffee are well documented, individual variability in responses necessitates more personalized studies. Several investigations suggest that regular coffee consumption is correlated with a reduced risk of age-related diseases, such as cardiovascular disease and certain cancers. However, the evidence is not uniformly conclusive across all cancer types. The anti-aging effects of coffee are likely associated with mitigating genomic instability and enhancing cellular repair mechanisms. We propose that conducting long-term studies to monitor the effects of coffee consumption on aging markers in diverse populations could yield more definitive evidence. Furthermore, identifying the specific bioactive compounds in coffee and elucidating their molecular interactions would help clarify their roles in aging processes. Exploring how genetic variations influence individual responses to coffee could also lead to more personalized dietary recommendations.

By addressing these research priorities, scientists can better harness coffee’s potential to improve health and quality of life while mitigating the challenges associated with its consumption. Coffee’s story, rooted in centuries of cultural and scientific significance, continues to evolve as a key element in modern nutrition and health sciences.

## Figures and Tables

**Table 1:** Antiaging and anticancer activity of coffee compounds.

Study Type	Model	Key Findings	References
Antiaging activity			
in vivo	*S. cerevisiae*	Coffee enhanced the viability of *S. cerevisiae*.	[95,146,147]
in vivo	*C. elegans*	Caffeine extended the lifespan of *C. elegans*, delayed larval development, and reduced reproduction and body length.	[137]
in vivo	*C. elegans*	Caffeine extended the lifespan of *C. elegans* without affecting food intake or reproduction. This effect was dependent on the IGF-1 pathway.	[138]
in vivo	*C. elegans*	Low concentrations of caffeine had a protective role in aging-related disorders.	[143]
in vivo	*C. elegans*	Prolonged caffeine consumption in *C. elegans* mitigated intestinal aging by modulating vitellogenesis.	[144]
in vivo	*C. elegans*	Kahweol, a major diterpene in coffee, extended the lifespan of *C. elegans* via the IGF-1 pathway.	[145]
in vivo	*C. elegans*	CGA prolonged the adult mean lifespan of N2 *C. elegans.*	[146]
in vivo	*D. melanogaster*	The anti-aging and immunosuppressive effects of caffeine observed in *D. melanogaster* larvae were consistent with those reported in mammalian model systems.	[170]
in vivo	*D. melanogaster*	The lifespan of *D. melanogaster* treated with 0.016 mM caffeine was significantly extended, likely due to the induction of the endogenous antioxidant genes *SOD1* and *CAT*.	[171]
in vivo	Mice (C57 BL/6 NCr)	Both regular and decaffeinated coffee consumption reduced hepatic total mTOR and phosphorylated mTOR levels in aged mice, independent of the Akt and AMP-activated protein kinase pathways.	[172]
in vivo	Mice (SAMP8)	Mice fed with coffee polyphenols (including CGA) and milk fat globule membrane exhibited increased survival rates and improved long-term memory retention.	[173]
Anticancer activity			
in vitro	Breast cancer: MCF-7, MDA-MB-231 cells	Caffeine decreased cell viability by promoting both apoptosis and necrosis.	[174]
in vitro	Squamous carcinoma: HN5, KYSE30 cells	Caffeine exhibited an inhibitory effect, leading to a reduction in the proliferation rate of cells.	[175]
in vitro	Prostate cancer: PC-3 cells	Caffeine affected cell mortality in a dose-dependent manner.	[176]
in vitro	Hepatocellular carcinoma: SMMC-7721 and Hep3 cells	Caffeine decreased the viability.	[177]
in vitro	Melanoma: B16F10 cells	Caffeine improved the cytotoxic effect of dacarbazine on B16F10 murine melanoma cells.	[178]
in vivo	Fibrosarcoma adult albino mice	Caffeine enhanced anti-tumor immune response.	[179]

## Data Availability

Not applicable.

## Abbreviations

AMP—adenosine monophosphate; ARE—antioxidant response element; CAT—catalase; CGA—chlorogenic acid; GPx—glutathione peroxidase; IGF-1—insulin-like growth factor 1; 3-NT—3-nitrotyrosine; MDA—malondialdehyde; mTOR—mammalian target of rapamycin; Nrf2—nuclear factor erythroid 2-related factor 2; OS—oxidative stress; ROS—reactive oxygen species; SOD—superoxide dismutase; TAC—total antioxidant capacity.
